# Targeted truncation of the ADAM17 cytoplasmic domain in mice results in protein destabilization and a hypomorphic phenotype

**DOI:** 10.1016/j.jbc.2021.100733

**Published:** 2021-05-04

**Authors:** Jose Lora, Gisela Weskamp, Thomas M. Li, Thorsten Maretzky, Dorjee T.N. Shola, Sébastien Monette, Stefan F. Lichtenthaler, Theresa T. Lu, Chingwen Yang, Carl P. Blobel

**Affiliations:** 1 Physiology, Biophysics and Systems Biology Program, Weill Cornell Medicine, New York, New York, USA; 2 Arthritis and Tissue Degeneration Program, Hospital for Special Surgery, New York, New York, USA; 3 Autoimmunity and Inflammation Program, Hospital for Special Surgery, New York, New York, USA; 4 Inflammation Program and Department of Internal Medicine, Roy J. and Lucille A. Carver College of Medicine, University of Iowa, Iowa City, Iowa, USA; 5 CRISPR and Genome Editing Resource Center, Rockefeller University, New York, New York, USA; 6 Tri-Institutional Laboratory of Comparative Pathology, Sloan-Kettering Institute, New York, New York, USA; 7 German Center for Neurodegenerative Diseases (DZNE), Technical University of Munich, Munich, Germany; 8 Neuroproteomics, School of Medicine, Klinikum rechts der Isar, Technical University of Munich, Munich, Germany; 9 Munich Cluster for Systems Neurology (SyNergy), Technical University of Munich, Munich, Germany; 10 Institute for Advanced Study, Technical University of Munich, Garching, Germany; 11 Department of Microbiology and Immunology, Weill Cornell Medicine, New York, New York, USA; 12 Department of Medicine, Weill Cornell Medicine, New York, New York, USA; 13 Department of Biophysics, Physiology and Systems Biology, Weill Cornell Medicine, New York, New York, USA

**Keywords:** ADAM17 (a disintegrin and metalloprotease 17), TACE (TNFα converting enzyme), cell surface enzyme, membrane protein, protein stability, myeloid cell, knock-in mutation in mice, ADAM17, a disintegrin and metalloprotease 17, BMDM, bone-marrow-derived macrophage, CHX, cycloheximide, DV, donor vector, EGFR, epidermal growth factor receptor, EPCAM, epithelial cell adhesion molecule, IL-6R, interleukin-6 receptor, iRhom1 and 2, inactive Rhomboid proteins 1 and 2, KI, knock-in, mEF, mouse embryonic fibroblast, mES, mouse embryonic stem, MFI, mean fluorescence intensity, PMA, phorbol 12-myristate 13-acetate, TNFα, tumor necrosis factor α

## Abstract

A disintegrin and metalloprotease 17 (ADAM17) is a cell-surface metalloprotease that serves as the principle sheddase for tumor necrosis factor α (TNFα), interleukin-6 receptor (IL-6R), and several ligands of the epidermal growth factor receptor (EGFR), regulating these crucial signaling pathways. ADAM17 activation requires its transmembrane domain, but not its cytoplasmic domain, and little is known about the role of this domain *in vivo*. To investigate, we used CRISPR-Cas9 to mutate the endogenous *Adam17* locus in mice to produce a mutant ADAM17 lacking its cytoplasmic domain (*Adam17Δcyto*). Homozygous *Adam17Δcyto* animals were born at a Mendelian ratio and survived into adulthood with slightly wavy hair and curled whiskers, consistent with defects in ADAM17/EGFR signaling. At birth, *Adam17Δcyto* mice resembled *Adam17−/−* mice in that they had open eyes and enlarged semilunar heart valves, but they did not have bone growth plate defects. The deletion of the cytoplasmic domain resulted in strongly decreased ADAM17 protein levels in all tissues and cells examined, providing a likely cause for the hypomorphic phenotype. In functional assays, *Adam17Δcyto* mouse embryonic fibroblasts and bone-marrow-derived macrophages had strongly reduced ADAM17 activity, consistent with the reduced protein levels. Nevertheless, ADAM17Δcyto could be stimulated by PMA, a well-characterized posttranslational activator of ADAM17, corroborating that the cytoplasmic domain of endogenous ADAM17 is not required for its rapid response to PMA. Taken together, these results provide the first evidence that the cytoplasmic domain of ADAM17 plays a pivotal role *in vivo* in regulating ADAM17 levels and function.

Cell–cell interactions are crucial for the development and maintenance of multicellular organisms. Protein ectodomain shedding of membrane-anchored signaling molecules and their receptors is considered an important mechanism for regulating cell–cell communications (1, 2, 3, 4). A disintegrin and metalloprotease 17 (ADAM17, A17, also referred as tumor necrosis factor α (TNFα) converting enzyme or TACE) was first discovered as the cell-surface metalloprotease that releases TNFα from its membrane anchor to elicit proinflammatory responses (5, 6). In addition, ADAM17 is the principal physiological sheddase for several ligands of the epidermal growth factor receptor (EGFR) (3, 7, 8, 9), the interleukin-6 receptor (IL-6R) (10, 11), and other cytokines and receptors (1, 2, 3, 4). ADAM17 controls EGFR signaling during development (7, 9, 12, 13, 14, 15) and has a key role in maintaining the skin and intestinal barrier (16, 17, 18). However, ADAM17 can also contribute to the pathogenesis of EGFR-dependent cancers (1, 19, 20), pathological neovascularization (21), and the development of autoimmune diseases involving dysregulated TNFα release and IL-6 trans-signaling *via* the soluble IL-6R (10, 11, 22, 23).

ADAM17 can be rapidly and posttranslationally activated in response to various physiological stimuli and to treatment with the phorbol ester phorbol 12-myristate 13-acetate (PMA) in a mechanism that requires its transmembrane domain, but not its cytoplasmic domain (24, 25, 26, 27, 28). The transmembrane domain of ADAM17 interacts with the seven-membrane-spanning inactive Rhomboid proteins 1 and 2 (iRhom1 and 2), which are crucial for the maturation and function of ADAM17 (29, 30, 31, 32). The cytoplasmic domain of ADAM17 contains protein–protein interaction domains, phosphorylation sites, and signaling motifs that have been suggested to play important roles in its function, regulation, subcellular transport, and recycling (33, 34, 35, 36, 37, 38, 39, 40, 41, 42, 43). However, the functional significance of the cytoplasmic domain of ADAM17 *in vivo* has remained elusive. The goal of this study was to generate mice carrying a mutant form of endogenous ADAM17 that lacks its cytoplasmic domain, including all previously described signaling motifs, in order to learn more about the role of the cytoplasmic domain in regulating the function of ADAM17 *in vivo*.

## Results

### Generation and characterization of *Adam17Δcyto* mice

**Figure 1:**
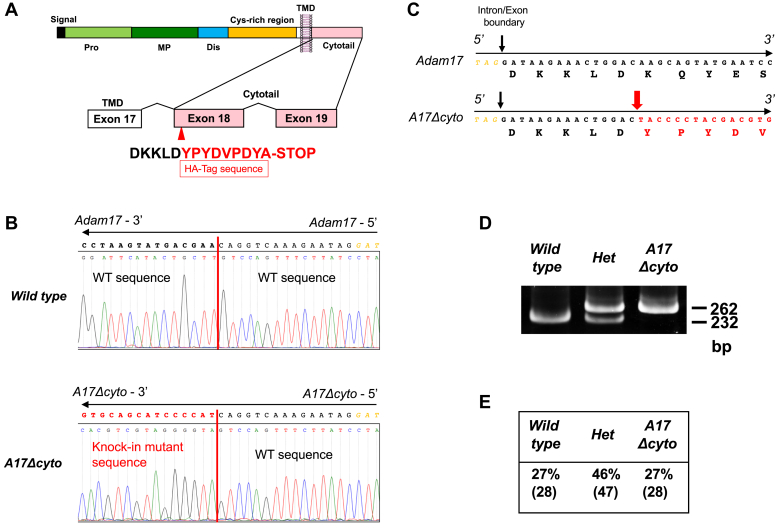
**Generation of *Adam17*Δ*cyto* mice and Mendelian distribution of offspring.***A*, diagram of the domain organization of ADAM17 (*top*) and of the corresponding transmembrane and cytoplasmic domain exons (*bottom*). A sequence coding for an HA-tag followed by a stop codon (*red capital letters*) was knocked-in adjacent to the cytoplasmic boundary of the transmembrane domain after the sequence DKKLD (*black capital letters*). *B*, genomic DNA sequence traces of the antisense strand of the targeted sequence in *Adam17Δcyto* mice and the corresponding *wild-type* sequence. The coding sequence is shown above the trace sequence going 5’ -> 3’ from *right* to *left*, starting from the intergenic triplet TAG-5’ in *black downstream* of the intron/exon boundary in *yellow*. The position of the targeted insertion site is indicated by a *vertical red line*, with the knock-in mutant sequence shown in *red capital letters* above the *Adam17Δcyto* sequence traces (*bottom panel*). *C*, the resulting genomic sequence of the *wild-type* locus or the targeted locus in *Adam17Δcyto* mice is shown in 5’ → 3’ orientation from *left* to *right* with the amino acid sequence translated from the coding region. *D*, PCR genotyping results generated from *wild-type*, *Adam17Δcyto* heterozygotes, and *Adam17Δcyto* homozygotic mutants. *E*, offspring from *Adam17Δcyto* het x het matings were born at an approximately Mendelian ratio.

In order to generate mice that express endogenous ADAM17 with a truncated cytoplasmic domain, we used CRISPR-Cas9 to introduce a targeted knock-in (KI) mutation, in which an HA-tag with a stop codon was attached immediately C-terminal to the transmembrane domain of ADAM17 (following DKKLD^699^, Fig. 1*A*, see Experimental procedures and Fig. S1 for details). The resulting mutant ADAM17Δcyto lacks almost all of the cytoplasmic domain of ADAM17 (cytoplasmic amino acid residues 700–827) and corresponds to an ADAM17Δcyto mutant that fully rescues ADAM17-dependent shedding when overexpressed in *Adam17−/−* mouse embryonic fibroblasts (mEFs) (27). Founder mice carrying the *Adam17Δcyto* KI mutation were generated following standard protocols (see Experimental procedures for details) and bred to homozygosity. The presence of the *Adam17Δcyto* mutation was verified by sequencing PCR fragments from genomic DNA of mutant mice compared with *wild-type* controls (Fig. 1, *B* and *C*). Offspring of heterozygous matings (*Adam17Δcyto/+ x Adam17Δcyto/+*) were genotyped by genomic PCR (Fig. 1*D*) and were born at the expected Mendelian ratio (Fig. 1*E*).

**Figure 2:**
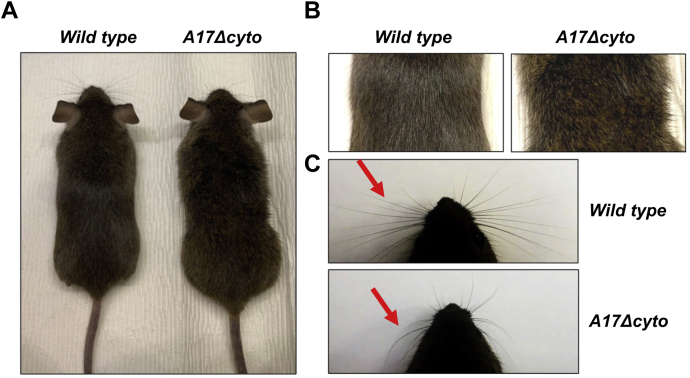
**Adult *Adam17*Δ*cyto* mice have wavy fur and curly whiskers.***A*, representative images of 6-month-old *wild-type* and *Adam17Δcyto* littermates show a wavy fur phenotype in the *Adam17Δcyto* mouse compared with the *wild-type* control. An enlarged image of the wavy fur in an adult *Adam17Δcyto* mouse is shown together with a *wild-type* control in (*B*). The curly whiskers in a 3-week-old mutant mouse are shown in comparison to straight whiskers in an age-matched *wild-type* mouse in (*C*). All images are representative samples for typical *wild-type* or *Adam17Δcyto* mice.

Homozygous mutant *Adam17Δcyto* animals appeared grossly normal (Fig. 2*A*) and did not display behavioral abnormalities during routine handling compared with *wild-type* controls. However, closer inspection revealed that *Adam17Δcyto* mice had slightly wavy fur (Fig. 2*B*) and curly whiskers (Fig. 2*C*, red arrows), which are characteristic phenotypes for mice carrying mutations affecting ADAM17/EGFR signaling (16, 44, 45).

**Figure 3:**
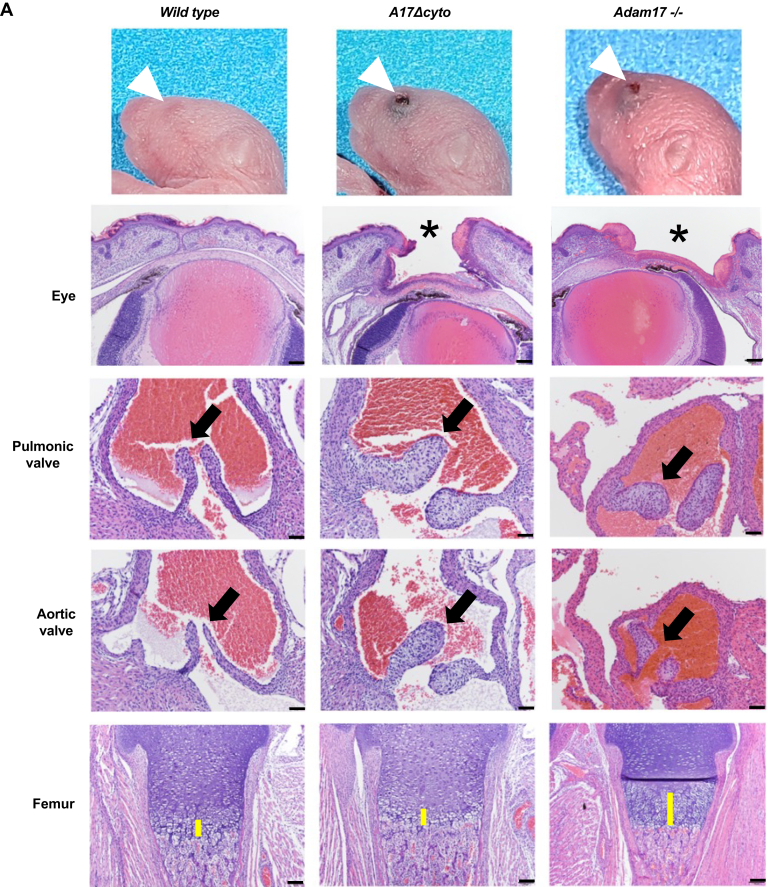

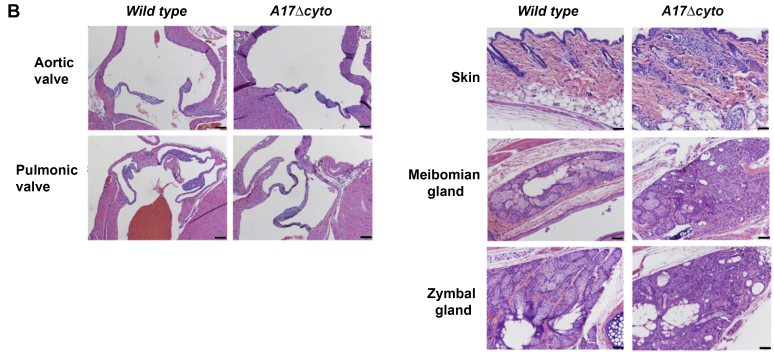
***Adam17*Δ*cyto* mice closely resemble *Adam17−/−* mice at birth.***A*, representative images of the heads of newborn *wild-type* and *Adam17Δcyto* (littermates) and *Adam17−/−* mice show open eyes at birth (OEB) in *Adam17Δcyto* mutants that are similar to the OEB in *Adam17−/−* mice (*top row*, *white arrows*). Corresponding H&E-stained sections of the eye are shown in row 2, with an *asterisk* marking the open eyelid. Sections of a representative pulmonic valve (*middle panel*/row 3) and aortic valve (row 4) show similarly enlarged and misshapen tricuspid valves in *Adam17Δcyto* mutants compared with *Adam17−/−* mice. However, the growth plate in the *Adam17Δcyto* mutant appeared normal in size and comparable to the *wild-type* control and did not display the enlarged zone of hypertrophic cells seen in *Adam17−/−* mice (*bottom row*). Scale bars: sections of eyes and femurs, 100 μm, sections of heart valves, 50 μm. *B*, representative images of the aortic and pulmonic valves in adult *Adam17Δcyto* mutants are indistinguishable from *wild-type* controls (*left panels*). The hair follicles and skin of *Adam17Δcyto* mutants showed pyogranulomatous inflammation, which also affected the meibomian glands in the eyelids and the zymbal glands near the ear canal (Fig. 3*B*, *right panels*). Scale bars: sections of adult heart valves, 100 μm, sections of skin and glands, 50 μm. All images are representative for sections from at least three mice per genotype.

Gross and histopathological analysis of newborn mice revealed that *Adam17Δcyto* animals resemble *Adam17−/−* animals in that their eyes are open at birth (Fig. 3*A*, top two rows of panels) (7, 46). Moreover, *Adam17Δcyto* mice show enlarged and thickened pulmonic, aortic, and tricuspid heart valves (Fig. 3*A*, middle and lower middle panels, tricuspid valve not shown), similar to *Adam17−/−* mice (12, 46). However, unlike *Adam17−/−* mice (14, 15), *Adam17Δcyto* mice did not have significantly expanded zones of hypertrophic cells in their long bone growth plate (Fig. 3*A*, femur shown in lower panel).

Analysis of adult *Adam17Δcyto* animals showed no evident abnormalities in heart weight and gross and histologic morphology of the heart, including the heart valves (Fig. 3*B*, left panels), suggesting that the heart valve defects are remodeled and return to normal as these animals grow into adults. In addition, we found pyogranulomatous inflammation of hair follicles in the skin and in the meibomian glands in eyelids and zymbal glands near the ear canal, which are both specialized sebaceous glands (Fig. 3*B*, right panels). There were no other major evident pathological phenotypes in the adult *Adam17Δcyto* mice compared with their littermate controls.

### Strong reduction in ADAM17 protein levels in *Adam17Δcyto* mouse embryonic fibroblasts

**Figure 4:**
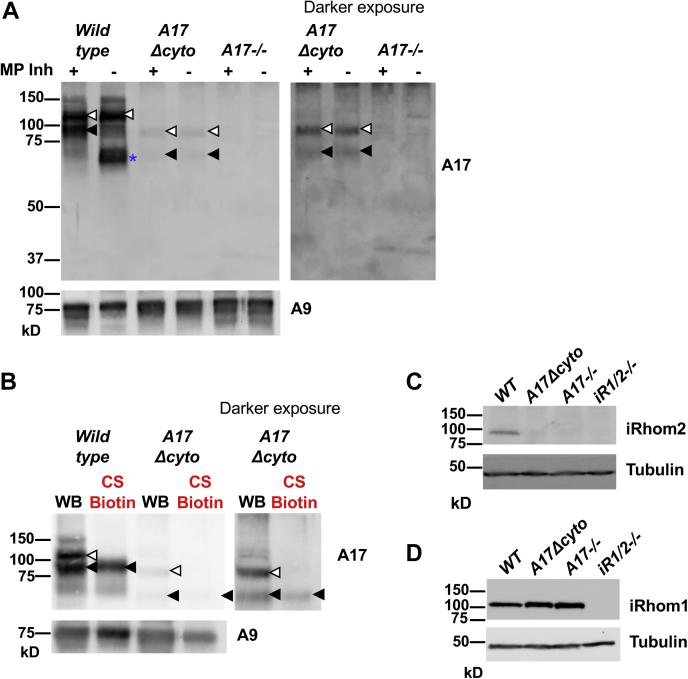
**Strongly reduced levels of endogenous ADAM17Δcyto protein in mouse embryonic fibroblasts.***A*, mouse embryonic fibroblasts (mEFs) were lysed in the presence or absence of the metalloprotease (MP) inhibitors marimastat (5 μm) and 1,10 Phenanthroline (10 mM), as indicated. Concanavalin A-enriched glycoproteins were subjected to a western blot analysis with anti-ADAM17-ecto antibodies, which showed strongly reduced expression of endogenous ADAM17Δcyto protein compared with wild-type ADAM17, with samples from *Adam17−/−* mEFs serving as negative control for the selectivity of the antibodies and ADAM9 (A9) as a loading control. The *right panel* shows a longer exposure of the *Adam17Δcyto* and *Adam17−/−* samples to better visualize the pro- and mature forms of ADAM17Δcyto. The postlysis autocatalytic degradation of mature wild-type ADAM17 in the absence of metalloprotease (MP) inhibitors can be seen in lane 2 of the wild-type sample (47, 48). *B*, western blot analysis (WB) compared with cell surface Biotin labeling (CS biotin) of ADAM17 and ADAM17Δcyto (see Experimental procedures for details). An *open arrowhead* indicates the proform, and a *black arrowhead* indicates the mature form of endogenous wild-type ADAM17 or ADAM17Δcyto. *C* and *D*, whole-cell lysates of *wild-type*, *Adam17Δcyto*, *Adam17−/−*, and *iRhom1/2−/−* mEFs were subjected to western blot analysis using monoclonal rat-antibodies against iRhom2 (*C*, *top panel*) or iRhom1 (*D*, *top panel*) or antibodies against alpha-tubulin (*lower panels* in *C* and *D*) as a loading control. The levels of iRhom2 were strongly reduced in *Adam17Δcyto*, just like in *Adam17-/-* mEFs, whereas the iRhom1 levels were slightly increased in *Adam17Δcyto* mEFs, as in *Adam17−/−* mEFs (50). Western blots for tubulin served as loading control, and lysates of *iRhom1/2−/−* mEFs served as reagent controls for the selectivity of the rat anti-iRhom1 or iRhom2 monoclonal antibodies. Each experiment was repeated three times with essentially similar results and one representative blot is shown.

The hypomorphic phenotype of *Adam17Δcyto* mice raised questions about the underlying cause for the apparently reduced activity of ADAM17. We therefore performed western blots on Concanavalin A-enriched glycoproteins isolated from *wild-type*, *Adam17Δcyto*, and *Adam17−/−* mEFs with rabbit polyclonal antibodies raised against the extracellular domain of mouse ADAM17 (anti-A17-ecto). The samples were run under denaturing, but nonreducing conditions, since the anti-A17-ecto antibodies only recognize nonreduced ADAM17 in western blots (see Fig. S2 and Experimental procedures for details). Under these conditions, the anti-A17-ecto antibody detected the wild-type proform of ADAM17 (Fig. 4*A*, indicated by an open arrowhead) as well as the mature form (indicted by a black arrowhead) in mEFs. By comparison, the levels of pro-ADAM17Δcyto (open arrowhead) and mature ADAM17Δcyto (black arrowhead) were strongly reduced. When *wild-type* cells were lysed in the absence of metalloprotease inhibitors (marimastat and 1,10 Phenanthroline, see Experimental procedures for details), the mature form of wild-type ADAM17 underwent postlysis autocatalytic removal of the cytoplasmic domain, as reported previously (47, 48), which generates a faster migrating form of mature ADAM17 (Fig. 4*A*, indicated by an asterisk), whereas pro-ADAM17 was not affected. A similar postlysis autocatalytic removal of the cytoplasmic domain was also observed in the related ADAM10 and is thought to have no biological significance in ADAM17 or ADAM10 (49). The proform of ADAM17Δcyto (open arrowhead in Fig. 4*A*, darker exposure shown on the right) migrated faster than the proform of wild-type ADAM17, consistent with the predicted reduction in molecular weight caused by deletion of the cytoplasmic domain. The mature form of ADAM17Δcyto (black arrowhead in Fig. 4*A*) comigrated approximately with the autodegraded form of mature wild-type ADAM17 (asterisk). However, the presence or absence of the metalloprotease inhibitors during cell lysis did not affect the migration of mature ADAM17Δcyto. This suggests that ADAM17Δcyto was not detectably subjected to postlysis processing, presumably because it lacks the cytoplasmic domain that is removed by this process in wild-type ADAM17. The samples from *Adam17−/−* mEFs served as negative control for the selectivity of the anti-A17-ecto antibodies.

Cell surface biotinylation followed by western blot analysis of streptavidin-purified biotinylated proteins showed that only the mature forms of wild-type ADAM17 and of ADAM17Δcyto (black arrowheads) were present at the cell surface (Fig. 4*B*, darker exposure for ADAM17Δcyto shown in the right panel). A densitometric comparison of cell surface biotinylated mature ADAM17 to the total ADAM17 detected by western blot showed a similar ratio for the wild-type and ADAM17Δcyto proteins (ratio of biotinylated ADAM17 to total ADAM17, wild-type average 0.46 arbitrary units, SEM 0.013; ADAM17Δcyto average 0.49 arbitrary units, SEM 0.094, Student’s *t*-test *p*-value 0.78, see Experimental procedures for details). Taken together, these results demonstrated that the deletion of the cytoplasmic domain of endogenous ADAM17 affects its levels in mEFs, but that the ADAM17Δcyto protein is nevertheless processed in the secretory pathway and transported to the cell surface.

Previous studies have shown that the presence of ADAM17 stabilizes its regulatory binding partner iRhom2, but not iRhom1 (50). Consistent with this observation, we found very low levels of iRhom2 in *Adam17Δcyto* mEFs, similar to *Adam17−/−* mEFs (Fig. 4*D*). The levels of iRhom1 appeared slightly increased in *Adam17Δcyto* mEFs, just like those observed in *Adam17−/−* mEFs (50). These findings further corroborate the dependence of iRhom2, but not iRhom1 on the levels of ADAM17.

### Strongly reduced ADAM17 protein levels in *Adam17Δcyto* mouse tissues

**Figure 5:**
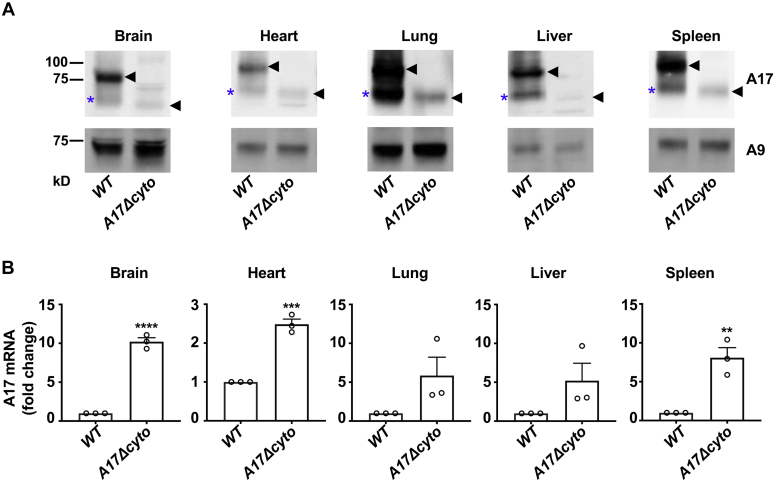
**Reduced levels of ADAM17Δcyto protein but not mRNA in selected tissues of adult *Adam17Δcyto* mice.***A*, representative western blots of extracts from the brain, heart, lung, liver, and spleen of adult mice show a strong reduction in ADAM17Δcyto protein levels compared with wild-type controls. *B*, *Adam17* mRNA expression in the brain, heart, lung, liver, and spleen of adult *wild-type* and *Adam17Δcyto* mice (n = 3, Student’s *t*-test, ∗ indicates a *p*-value of <0.05). Each western blot is representative for at least three separately prepared western blots, each from mice from a different litter. *Black arrowhead*, mature wild-type or ADAM17Δcyto, as indicated, *blue asterisk*, postlysis autodegradation artifact of mature wild-type ADAM17. Western blots for ADAM9 are shown as loading control.

**Figure 6:**
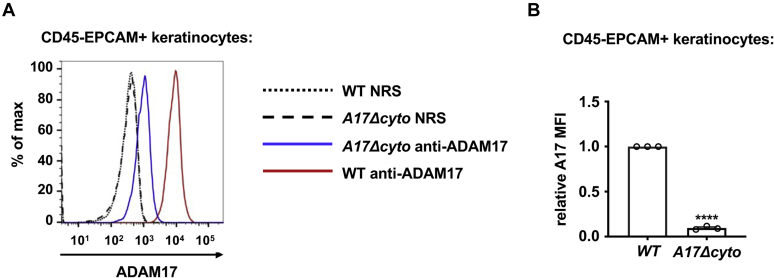
**Flow cytometric analysis of ADAM17 levels on the surface of cells isolated from mouse skin.** Cell surface ADAM17 levels on *ex vivo* keratinocytes. Ears from *wild-type* or *Adam17Δcyto* mice were digested to single cell suspensions, stained with anti-ADAM17 or normal rabbit serum (NRS) control along with cellular markers, and subjected to flow cytometric analysis. *A*, histograms of ADAM17 levels for live-gated CD45- EPCAM+ cells (mostly keratinocytes). *B*, mean fluorescence intensity (MFI) of ADAM17 signal normalized to the signal in the *wild-type* sample for each experiment. A representative example from three separate experiments is shown in *A*, each symbol represents one mouse in *B*. ∗ indicates a *p*-value of <0.05 in an unpaired Student’s *t*-test.

As a next step, we performed western blots with anti-A17-ecto on extracts from the brain, heart, lung, liver, and spleen tissue of *wild-type* and *Adam17Δcyto* mice. This corroborated that the levels of both pro- and mature ADAM17 were strongly reduced in these tissues in *Adam17Δcyto* mice compared with controls (Fig. 5*A*, black arrowhead indicates mature wild-type ADAM17, or ADAM17Δcyto, the proform of ADAM17 is not detected efficiently by the anti-A17-ecto antibodies in tissues, please see Fig. S2 for details). In some cases, the *wild-type* samples contained a small amount of the autodegraded mature form of ADAM17 (asterisk in Fig. 5*A* (47, 48)). An analysis of the mRNA levels of *wild-type* or mutant *Adam17* by qPCR in the lung and liver showed an upward, but not significant trend in mRNA levels, whereas there was a significant increase in the expression of *Adam17 mRNA* in *Adam17Δcyto* brain, heart, and spleen compared to *wild-type* controls (Fig. 5*B*). When we performed flow cytometry analysis of skin cells that were positive for the epithelial cell adhesion molecule (EPCAM+, mostly keratinocytes), we saw a strong reduction in surface ADAM17 signal in cells from *Adam17Δcyto* mice compared with *wild-type* controls (Fig. 6, *A* and *B*). Taken together, these findings emphasize that the truncation of the cytoplasmic domain of endogenous ADAM17 strongly affects the levels of ADAM17Δcyto in different tissues and cells in mice, most likely in a posttranslational manner.

### Activity of ADAM17Δcyto in mEFs

**Figure 7:**
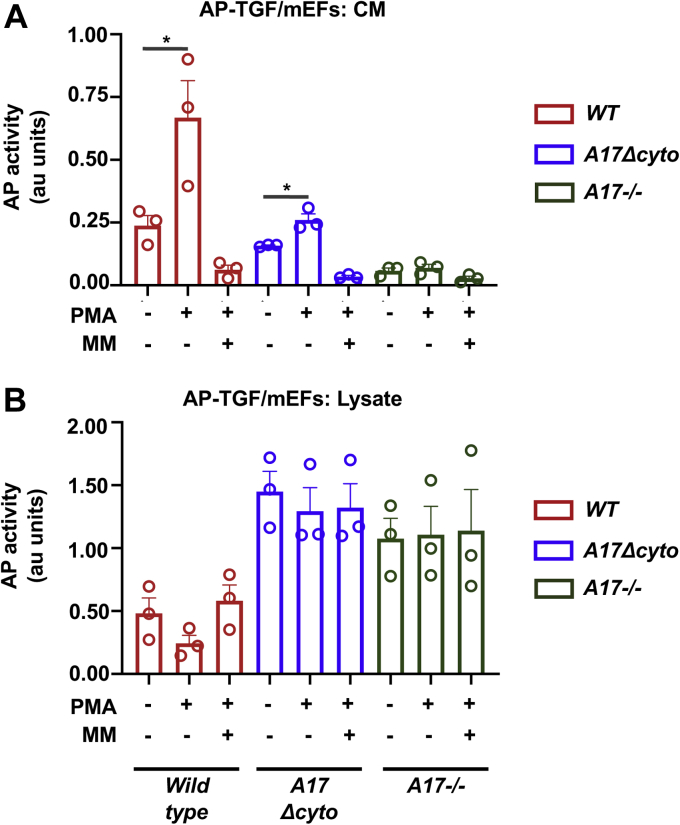
***Adam17Δcyto* mEFs can support constitutive and PMA-stimulated shedding of the ADAM17 substrate TGFα at overall reduced levels.** mEFs from *wild-type*, *Adam17Δcyto*, and *Adam17−/−* mice were transfected with the alkaline phosphatase-tagged ADAM17-selective substrate TGFα (TGFα-AP) and left untreated or stimulated with 25 ng/ml PMA for 45 min in the presence or absence of 5 μM of the metalloprotease inhibitor marimastat. *A*, the activity of ADAM17 and ADAM17Δcyto is shown as the TGFα-AP activity in the conditioned media (CM). ADAM17Δcyto supports overall lower levels of TGFα-AP shedding, but can respond to stimulation with PMA. *Adam17−/−* mEFs serve as a negative control. *B*, the reduced activity of ADAM17Δcyto compared with wild-type ADAM17 is also reflected in the higher levels of TGFα-AP in the lysates of *Adam17Δcyto* cells, similar to *Adam17−/−* mEFs, presumably caused by the reduced overall shedding activity. n = 3. ∗ indicates a *p*-value of <0.05 in an unpaired Student’s *t*-test.

Next, we tested how the substantially reduced levels of ADAM17Δcyto in *Adam17Δcyto* mEFs affected the shedding of the ADAM17-substrate TGFα. We found that PMA-stimulated processing of TGFα was strongly reduced in *Adam17Δcyto* mEFs compared with *wild-type* controls (Fig. 7*A*), presumably caused by the strong decrease in ADAM17Δcyto protein levels (see Fig. 4). Nevertheless, the shedding of TGFα was significantly enhanced by addition of the phorbol ester phorbol 12-myristate 13-acetate (PMA) in *Adam17Δcyto* mEFs (Fig. 7*A*), although the fold increase in shedding upon addition of PMA was reduced. Specifically, the PMA-stimulated increase in TGFα shedding in *wild-type* mEFs was on average 3.37-fold over the shedding levels in untreated controls (SEM 0.34), whereas *Adam17Δcyto* mEFs showed an average of 1.86-fold increase upon PMA stimulation (SEM 0.28). These results are consistent with previous studies that used overexpressed ADAM17Δcyto in that the cytoplasmic domain of endogenous ADAM17 is not required for its rapid activation following PMA stimulation (27), although the fold increase is lower than that for wild-type ADAM17. The strong reduction in shedding in the *Adam17Δcyto* mEFs was also reflected in the increased levels of presumably unprocessed TGFα-AP in the cell lysates, which were similar to the levels in *Adam17−/−* mEFs (Fig. 7*B*).

### Characterization of ADAM17Δcyto levels and function in bone-marrow-derived macrophages (BMDMs)

**Figure 8:**
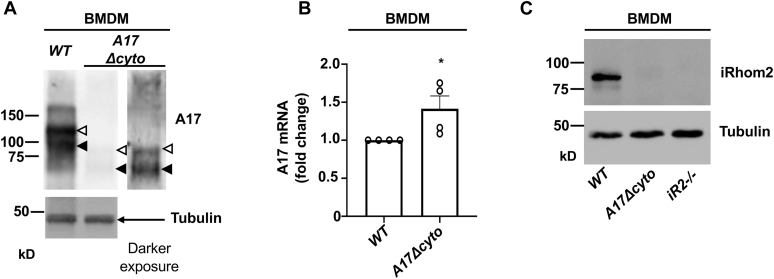
**Characterization of ADAM17Δcyto and iRhom2 expression in bone-marrow-derived macrophages (BMDMs).***A*, western blots of *wild-type* and *Adam17Δcyto* BMDMs probed with anti-A17-ecto antibodies show strongly decreased levels of ADAM17Δcyto compared with wild-type ADAM17. The proform of wild-type ADAM17 or ADAM17Δcyto is indicated by an *open arrowhead* and the mature form of each by a *black arrowhead*. The *inset* shows a darker exposure of the ADAM17Δcyto lane, and anti-alpha tubulin Westerns served as loading controls. *B*, a qPCR analysis showed slightly, but significantly increased expression of *Adam17Δcyto* mRNA compared with the *wild-typeAdam17* mRNA controls. *C*, a Western blot for iRhom2 showed barely detectable levels in *Adam17Δcyto* BMDMs compared with *wild-type* controls, with *iRhom2−/−* BMDMs serving as reagent control for the anti-iRhom2 antibody, and α-tubulin serving as a loading control. The western blots in panels *A* and *C* are representative of at least three separate experiments.

Previous studies have shown that the function of ADAM17 depends on iRhom2 in cells of myeloid origin, raising questions about how the truncation of the ADAM17 cytoplasmic domain impacts its levels and activity in these cells. We found strongly reduced levels of pro and mature ADAM17Δcyto in bone-marrow-derived macrophages (BMDMs) from *Adam17Δcyto* mice compared with controls (Fig. 8*A*), consistent with the reduction of ADAM17Δcyto protein levels observed in mEFs and adult tissues. A qPCR analysis showed that the mRNA levels for *Adam17Δcyto* are slightly but significantly higher than the mRNA levels for *Adam17* in *wild-type* BMDMs (Fig. 8*B*). We also found that iRhom2 cannot be detected in *Adam17Δcyto* BMDMs under conditions where it is easily detected in *wild-type* controls (Fig. 8*C*), just as in mEFs (see above), with *iRhom2−/−* BMDMs serving as control for the specificity of the anti-iRhom2 antibodies.

**Figure 9:**
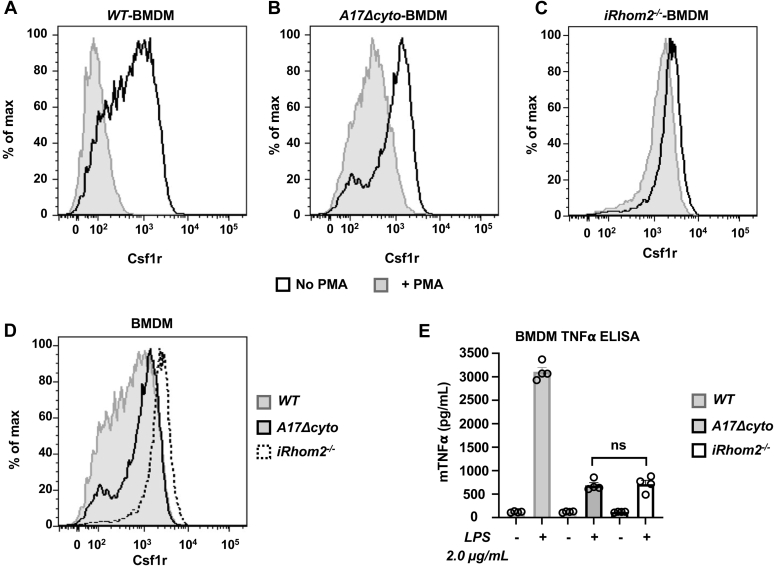
**Characterization of the activity of ADAM17Δcyto in bone-marrow-derived macrophages (BMDMs).***A*–*D*, cell surface levels of the iRhom2/ADAM17-substrate colony stimulating 1 receptor (Csf1R) were determined by flow cytometry in *wild-type*, *Adam17Δcyto*, and *iRhom2−/−* BMDMs, which served as negative control because they lack functional ADAM17 at the cell surface (23, 48, 54). *A*–*C*, stimulation of BMDMs with the phorbol ester PMA (25 ng/ml for 45 min), which activates ADAM17, strongly reduced the levels of the Csf1R on *wild-type* BMDMs (*A*) and led to a substantial, but somewhat weaker reduction on *Adam17Δcyto* BMDMs (*B*), with *iRhom2−/−* serving as control for the minor shift seen in the absence of ADAM17 (*C*). In unstimulated BMDMs, the levels of the Csf1R were lower in *Adam17Δcyto* compared with *iRhom2−/−* BMDMs, but higher than in the *wild-type* controls, consistent with reduced activity in *Adam17Δcyto* BMDMs (*D*). *E*, a TNFα ELISA on the supernatants of BMDMs treated with or without 2 μg/ml LPS for 3 h showed strongly reduced stimulation of TNFα production in *Adam17Δcyto* and *iRhom2−/−* BMDMs compared with *wild-type* controls, with no significant difference between *Adam17Δcyto* and *iRhom2−/−* BMDMs (Two-Way ANOVA, n = 4). Each flow cytometry trace is representative of at least three separate experiments. The FACS histograms in *A*–*D* show the results of one single representative experiment presented in different combinations, as indicated.

To measure ADAM17-dependent shedding of an endogenous substrate in *Adam17Δcyto* BMDMs, we monitored the cell surface levels of colony stimulating factor 1 receptor (Csf1r) *via* flow cytometry (51, 52, 53). Stimulation of BMDMs with PMA triggered a strong reduction in cell surface Csf1r levels in *wild type* and in *Adam17Δcyto* BMDMs compared with untreated controls (Fig. 9, *A* and *B*), although the shift was weaker in the *Adam17Δcyto* cells. There was only a slight drop in Csf1r levels in PMA-treated *iRhom2−/−* BMDMs compared with untreated controls, as described previously (Fig. 9*C*) (52). In nonstimulated cells, the surface levels of Csf1r were slightly lower in *Adam17Δcyto* BMDMs compared with *iRhom2−/−* BMDMs, which have no detectable mature and active ADAM17 on their surface (48, 54), but substantially higher than in *wild-type* controls (Fig. 9*D*). These results provide independent verification that ADAM17-dependent shedding is reduced in *Adam17Δcyto* BMDMs, but that endogenous ADAM17Δcyto activity can nevertheless be enhanced by stimulation with PMA.

Since ADAM17 is the major sheddase for TNFα (5, 6, 7, 46), we also compared the LPS-stimulated release of TNFα from *wild-type*, *Adam17Δcyto*, and *iRhom2−/−* BMDMs. We found that stimulation of *wild-type* BMDMs with LPS for 3 h resulted in strongly induced levels of TNFα, whereas the LPS stimulation of *Adam17Δcyto* BMDMs yielded a similar increase in TNFα shedding as in LPS-stimulated *iRhom2−/−* BMDMs (Fig. 9*E*). Since ADAM10 is also able to cleave TNFα (55, 56), the similar residual TNFα shedding activity in *Adam17Δcyto* and in *iRhom2−/−* BMDMs presumably depends on ADAM10, which might obscure the relatively low activity of ADAM17Δcyto under these conditions. However, it should nevertheless be emphasized that ADAM17 and not ADAM10 is the principal TNFα convertase in the context of endotoxin shock *in vivo* (46). These results provide additional evidence for the substantial reduction of ADAM17 activity in *Adam17Δcyto* BMDMs compared with *wild-type* controls.

### Effect of protein translation and degradation inhibitors on ADAM17Δcyto levels

**Figure 10:**
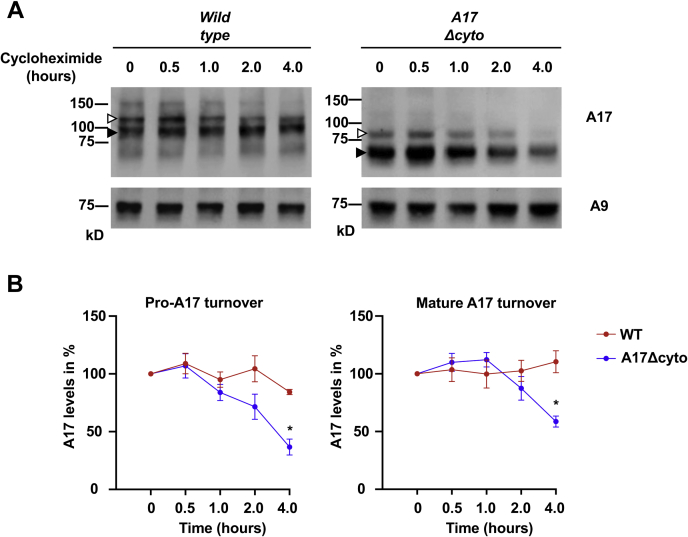
**Cycloheximide chase experiment of wild-type ADAM17 and the ADAM17Δcyto mutant in mEFs.** mEFs from *wild-type* or *Adam17Δcyto* mice were subjected to 100 μg/ml cycloheximide treatment (CHX) and chased for 4 h to compare how this affects the levels of wild-type ADAM17 and mutant ADAM17Δcyto protein at different time points (0’, 30’, 1 h, 2 h, 4 h). *A*, western blot analysis demonstrated that the levels of pro- and mature wild-type ADAM17 are not significantly affected under these conditions (*left panels*), whereas both the pro- and mature forms of ADAM17Δcyto show slightly reduced levels after 2 h CHX treatment and strongly reduced levels after 4 h in CHX. *Open arrowheads*, proform, *black arrowheads*, mature form of ADAM17, or ADAM17Δcyto. The western blot of ADAM17Δcyto was exposed longer than the blot of wild-type ADAM17 to allow a better comparison of the relative levels at different time points compared with untreated cells, with ADAM9 serving as a loading control (*lower panel*). *B*, densitometric analysis of the results of four separate experiments with NIH Image-J corroborated a significant decrease in pro- and mature ADAM17Δcyto compared with the *wild-type* control after 4 h in CHX. The graphs are based on four separate experiments, the 0 h time point is set to 100% for each experiment, and each data point represents the ADAM17 levels in percent relative to the 0 h time point for the proform or the mature form of ADAM17 or ADAM17Δcyto. To test for differences between *wild-type* and *Adam17Δcyto* at different time points, we performed an unpaired Student’s *t*-test with Welch correction and post hoc Bonferroni–Dunn correction for multiple hypothesis testing. ∗ indicates an adjusted *p*-value of <0.05.

To explore the causes of the reduced levels of ADAM17Δcyto protein, we performed a cycloheximide (CHX) chase experiment in mEFs. Western blots for ADAM17 or ADAM17Δcyto were performed at different time points after addition of this inhibitor of protein translation (Fig. 10). Under these conditions, the levels of pro- and mature ADAM17Δcyto were slightly reduced compared with the wild-type control after 2 h of CHX treatment and significantly reduced after 4 h in CHX. There was no significant change in the levels of pro- or mature ADAM17 compared with the untreated cells (0 h) at any of these time points (Fig. 10*B*), consistent with the results of a previous pulse chase experiments with radioactively labeled wild-type ADAM17 (47).

**Figure 11:**
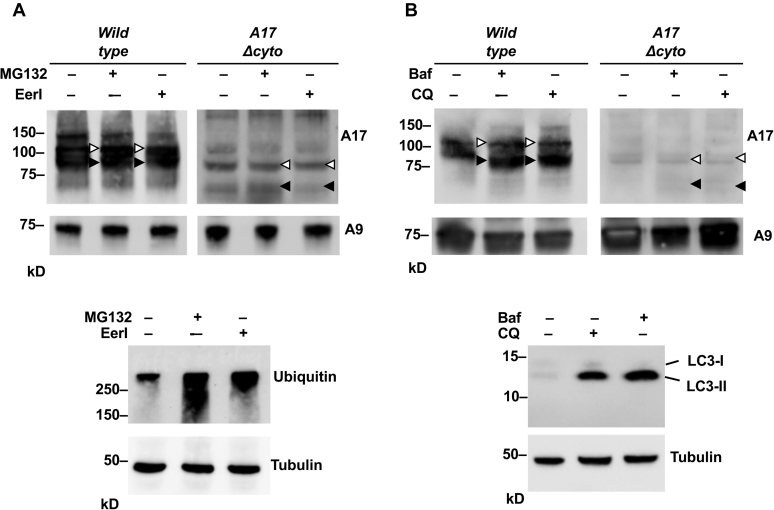
**Effect of inhibitors of proteasomal, lysosomal, and ER-associated degradation on ADAM17 levels in *wild-type* and *Adam17Δcyto* cells.***Wild-type* or *Adam17Δcyto* mEFs were either treated with 10 μM of the proteasomal inhibitor MG-132 or the ER-associated degradation (ERAD) inhibitor Eeyarestatin I (EerI) for 6 h or left untreated before lysis (*A*). Concanavalin A-enriched protein extracts were subjected to western blot analysis for either ADAM17 or ADAM9 as a loading control (*A*). As a control for the activity of these inhibitors, lysates of *Adam17Δcyto* mEFs treated under identical conditions were subjected to western blot analysis for Ubiquitin, which corroborated the increased levels caused by a block of proteasomal degradation for MG-132 or of ERAD, with a blot for tubulin serving as a loading control (*lower panels*). In addition, *wild-type* or *Adam17Δcyto* mEFs were treated with 100 nM Bafilomycin (BAF) or 100 μM Chloroquine (CQ) for 18 h or left untreated and then lysed and prepared for western blot analysis for ADAM17 or ADAM9 as a loading control (*B*). The activity of inhibitors of lysosomal degradation was confirmed in western blots of extracts of *Adam17Δcyto* mEFs treated with Baf or CQ that were probed with antibodies against LC3-II, which accumulates upon inhibition of the lysosomal degradation pathway (*lower panels*). Please note that 10% Bis-Tris NuPage gels were used to separate the samples for the western blots shown for ubiquitin, LC3, and the tubulin controls in the lower panels and for the A9 control in *A*. The results are representative of three independent experiments with essentially similar findings.

Next, we examined whether inhibitors of lysosomal or proteasomal degradation could increase or restore the levels of ADAM17Δcyto in mEFs. When we incubated *Adam17Δcyto* mEFs with the proteasomal inhibitor MG-132 or the ER-associated degradation pathway inhibitor Eeyarestatin, no substantial changes in the levels of wild-type ADAM17 or of ADAM17Δcyto were detected by western blot analysis (Fig. 11*A*). Under these conditions, both inhibitors blocked ubiquitin degradation, which served as a control for their activity (Fig. 11*A*, bottom panels). Similar results were obtained when *Adam17Δcyto* mEFs were treated with the lysosomal degradation inhibitors chloroquine or bafilomycin, which also did not lead to a substantial increase in the levels of wild-type ADAM17 or ADAM17Δcyto (Fig. 10*B*). For both inhibitors, an increase in LC3-II served as an indicator of effective inhibition of autophagy under the conditions used here (Fig. 11*B*, lower panel). These results argue against a major role of lysosomal, proteasomal, or autophagosomal degradation in causing the low levels of ADAM17Δcyto.

## Discussion

The main goal of this study was to examine the function of the cytoplasmic domain of endogenous ADAM17 *in vivo*. Previous studies have reported that phosphorylation of the cytoplasmic domain of ADAM17 is required for its activation (40, 41, 42, 43), whereas several other studies reported that a mutant ADAM17 lacking its cytoplasmic domain could be stimulated as well as the wild-type control (24, 26, 27). On the other hand, the maturation of overexpressed wild-type ADAM17 differed from the endogenous wild-type protein in that very little of the mature form of overexpressed ADAM17 could be detected by western blot analysis (57). The different properties of overexpressed *versus* endogenous ADAM17 provided an obstacle in the analysis of how the cytoplasmic domain controls the maturation, localization, and function of ADAM17. To circumvent this problem, we used CRISPR-Cas9 to generate an endogenous ADAM17Δcyto mutant with the same amino acid sequence as the previously studied overexpressed ADAM17Δcyto (27). Here we report the analysis of the properties of the endogenously expressed ADAM17Δcyto in *Adam17*Δ*cyto* KI mice. We show that the levels of endogenous ADAM17Δcyto are strongly reduced compared with wild-type controls. Nevertheless, *Adam17Δcyto* mice survive and are viable and fertile, although they display hypomorphic ADAM17/EGFR phenotypes with open eyes and heart valve defects at birth and curly whiskers and wavy hair as adults. The hypomorphic phenotype of *Adam17Δcyto* mice is most likely a consequence of the low levels of ADAM17Δcyto protein.

To gain a better understanding of how the decreased levels of ADAM17Δcyto affect its function, we performed cell-based assays for the shedding of the ADAM17 substrate TGFα. We found that the strong reduction in ADAM17Δcyto protein levels was reflected by strongly reduced activity toward TGFα. Nevertheless, endogenous ADAM17Δcyto exhibited a significant response to stimulation with the phorbol-ester PMA, a commonly used activator of ADAM17-dependent shedding of substrates such as overexpressed TGFα or the endogenous Csf1r (9, 52, 53), although the level of the response was reduced. Since mature ADAM17Δcyto and wild-type ADAM17 can be detected on the cell surface at a similar relative level compared with the total protein detectable by western blot, these findings demonstrate that ADAM17Δcyto can undergo maturation and is transported to the cell surface, where it can respond rapidly to PMA stimulation. Previous studies have shown that phosphorylation of iRhom2 is crucial for the iRhom2-dependent activation of ADAM17 (29, 30, 31, 32). Moreover, several studies have shown that the stimulation of ADAM17 by iRhom2 requires sequences in the first transmembrane domain of iRhom2 and in the transmembrane domain of ADAM17 (53, 58, 59). Taken together, these studies help explain how ADAM17 can be activated in the absence of its cytoplasmic domain *via* phosphorylation of iRhom2, which interacts with ADAM17 *via* their transmembrane domains. The functional properties of ADAM17Δcyto therefore corroborate previous results with overexpressed ADAM17Δcyto (24, 26, 27) in that the cytoplasmic domain of endogenous ADAM17 is not required for its rapid posttranslational activation by PMA.

An unexpected finding of this study was that the lack of the cytoplasmic domain of ADAM17 led to strongly reduced levels of both pro- and mature ADAM17Δcyto, despite normal or even increased levels of *Adam17Δcyto* mRNA. The strong reduction in ADAM17Δcyto protein levels was seen by western blot in mEFs, BMDMs, and in different tissues in adult mice and by flow cytometry in skin cells, including keratinocytes. Despite the strongly reduced levels and activity of ADAM17Δcyto in cell-based assays, the remaining activity was sufficient to allow *Adam17Δcyto* mice to survive, whereas most *Adam17−/−* mice die shortly after birth (7, 46). However, since the *Adam17Δcyto* mice are born with open eyes and heart valve defects, like *Adam17−/−* mice, this suggests that the normal development of these structures is sensitive to the levels and activity of ADAM17 during development. The heart valve defects were no longer apparent in adult *Adam17Δcyto* mice, suggesting that these defects can be repaired or remodeled after birth, although we cannot rule out that the adult animals analyzed had milder heart valve defects at birth.

The curved whiskers, wavy fur, and inflamed Meibomian glands in adult mice are consistent with a reduction in ADAM17/TGFα/EGFR signaling (45, 60, 61), which presumably also explains the inflammation of Zymbal glands. With respect to the curvy whiskers and wavy fur, the *Adam17Δcyto* mice resemble other mice with hypomorphic mutations in the ADAM17/EGFR pathways (16, 44, 45). The ability of female *Adam17cyto* mice to give birth and nurse their offspring suggests that reproductive functions and lactation, both of which are known to involve EGFR signaling and ADAM17-dependent processing of the EGFR ligand Amphiregulin (13, 62, 63), are not strongly affected.

Previous studies have shown that pro-ADAM17 is synthesized and retained in the ER in the absence of iRhom1 and 2, with no apparent effect on the stability of pro-ADAM17 (64, 65). The mutant ADAM17Δcyto protein was significantly less stable than the wild-type control in cycloheximide chase experiments in mEFs, demonstrating that the cytoplasmic domain of ADAM17 has an important role in regulating its stability and turnover at the protein level. The low levels of pro- and mature ADAM17Δcyto raise the possibility that a putative and yet-to-be identified stability factor binds the ADAM17 cytoplasmic domain to stabilize this protein until it can interact with an iRhom protein. Binding of an iRhom to ADAM17 in the ER would then initiate the maturation and trafficking of ADAM17 to the cell surface. A previous study showed that hardly any iRhom2 can be detected in the absence of ADAM17, whereas the levels of iRhom1 were slightly increased (50). Similar results were obtained in a western blot analysis of *Adam17Δcyto* mEFs, which had very little iRhom2 but slightly increased levels of iRhom1, as well as in *Adam17Δcyto* BMDM, which had very little iRhom2, providing additional corroboration that iRhom2 is stabilized by the presence of ADAM17. The levels of ADAM17Δcyto in mEFs were not strongly affected by treatment with inhibitors of proteasomal, lysosomal, or ER-associated degradation pathways, suggesting that these pathways do not play a major role in causing the reduced levels of ADAM17Δcyto.

Taken together, these findings are the first to identify a role of the cytoplasmic domain of ADAM17 in stabilizing the levels of this protein. Moreover, they confirm that the cytoplasmic domain of endogenous ADAM17 is not required for its rapid activation by PMA. The reduced levels of ADAM17 are sufficient to support survival of the *ADAM17Δcyto* mice, although they have several ADAM17/EGFR-dependent developmental defects, such as open eyes at birth and heart valve defects, demonstrating that the normal development of these structures requires higher or more physiological activity of ADAM17. Further studies will be necessary to understand the mechanism underlying the regulation of ADAM17 levels by its cytoplasmic domain.

## Experimental procedures

### Reagents and antibodies

Unless otherwise noted, all reagents were purchased from Sigma-Aldrich. The rabbit polyclonal antibody against the ectodomain of ADAM17 was generated by injecting a purified Fc fusion protein with the extracellular domain of human ADAM17 into New Zealand White rabbits (Covance). The rabbit polyclonal antibodies against ADAM9 and the rat monoclonal antibodies against iRhom1 and iRhom2 have been previously described (50, 66). The antibodies against alpha-tubulin were from Cell Signaling, (Cat. #: 2144). The antibody against LC3 was from Novus Biological (Cat. #: Nb.100-2220), and the anti-ubiquitin antibody was from Biolegend (Cat. # 838703). The cell surface biotin reagent EZ Link Sulfo-NHS-LC-Biotin was from Thermo Fisher Scientific (Cat. # 21335), as were the Streptavidin-Sepharose beads (Cat. #: 434341). The metalloprotease inhibitor marimastat was kindly provided by Dr Ouathek Ouerfelli, Memorial Sloan Kettering Cancer Center, New York, NY (67). Cycloheximide was obtained from Cell Signaling (Cat. #: 2112S). The inhibitors of proteasomal degradation, ER-associated degradation (ERAD), and lysosomal degradation were from Sigma-Aldrich: MG-132 (Cat. #: 474790), eeyarestatin (Cat. #: E1286), bafilomycin (Cat. #: B1793), and chloroquine (Cat. #: C6628).

### Mouse lines

The *Adam17−/−* and *iRhom2−/−* mice used in this study have been described previously (46, 48). To generate the *Adam17Δcyto* mice, a KI mutation was introduced by targeting the endogenous *Adam17* locus *via* CRISPR-Cas9 KI mutagenesis.

#### Construction of the *Adam17Δcyto* donor vector (DV)

The KI donor shown in Fig. S1*B* consists of an HA-tag with a stop codon flanked by 5’-homology sequence (514 bp) and 3’-homology sequence (587 bp) to the genomic target. The template was generated by PCR amplifications of two DNA segments from C57Bl/6J-TyrC-2J mouse genomic DNA using two pairs of primers that carry the sequences of HA-tag, stop codon, and BsaI restriction enzyme (RE) sites (see Table S1 in supporting information for details), followed by ligating the PCR products with a pFUS vector backbone *via* the Golden Gate cloning method (68). Additionally, the seed sequence of the CRISPR target site on the DV was mutagenized with primer Mut-F using QuickChange II (Agilent; Cat. #: 200521) to avoid recleavage of the repaired sequence post KI.

#### Selection of guide RNA

We identified a CRISPR target site (CCTATGCTTTCTAGGATAAGAAA; Fig. S1*A*, red scissors) at the junction of intron 17 and exon 18 for its optimal proximity to the DV insertion site, albeit with low off-target score. In order to mitigate off-target effects, a shorter version of single guide RNA (sgRNA) carrying only 17 nucleotides sequence upstream of the PAM (CTTATCCTAGAAAGCAT) was hence chosen (69) and was cloned into the pX330 vector (Addgene, plasmid # 42230; a SpCas9 and sgRNA expressing plasmid) following the protocol as described (70). The selected gRNA showed modest cleavage efficiency assessed in mouse embryonic stem (mES) cells.

#### Gene editing in mES cells

The DV (7 μg) and sgRNA+Cas9 expressing pX330 vector (2 μg) were transfected into C57Bl/6J-TyrC-2J mES cells (3 × 10^6^ cells) using Lipofectamine 2000 reagent (Thermo Fisher; Cat #: 11668019) with a modified transfection procedure for cell suspensions. Transfected ES cells were plated on mEF feeder cells at low density to obtain single colonies without selection, and individual ES colonies were isolated after 5 days of culture. Genomic DNA from the single colonies were screened *via* PCR using the external primer pair ScrA17-F and ScrA17-R, which amplified 1264 bp for the KI allele *versus* 1234 bp for the *wild-type* allele. The HA-tag carries a BmgBI site, which upon digestion cleaved the 1264 bp KI mutant into two fragments (659 bp and 605 bp), whereas the 1234 bp wild-type fragment was unaffected (Fig. S1*C*). The PCR product from the positive clone was identified through TOPO cloning as a mixed population, hence each colony was subjected to an additional round of clonal isolation, followed by PCR screening and Sanger sequencing validation. The edited ES cells were microinjected into C57Bl/6J blastocyst and implanted into pseudo-pregnant females. Chimera progeny was obtained and high degree chimeras were mated with C57Bl/6J-TyrC-2J mice to identify germline transmission of the *Adam17Δcyto* allele.

All animal experiments were approved by the Institutional Animal Care and Use Committee of the Hospital for Special Surgery and Weill Cornell Medicine.

### Necropsy and histopathology

The histopathological analysis of adult mice was performed on three pairs of mice from independent litters on a mixed 129SvJ/C57Bl/6J-TyrC-2J genetic background, each pair consisting of one *Adam17Δcyto* mutant and one *wild-type* littermate control. Two pairs were females and one pair was male. All mice were approximately 6.5 weeks old.

Mice were euthanized with CO_2_. Following gross examination, all organs were fixed in 10% neutral buffered formalin, followed by decalcification of bone in a formic acid solution (Surgipath Decalcifier I, Leica Biosystems). Tissues were then processed in ethanol and xylene and embedded in paraffin in a Leica ASP6025 tissue processor. Paraffin blocks were sectioned at 5 microns, stained with hematoxylin and eosin (H&E), and examined by a board-certified veterinary pathologist. The following tissues were processed and examined: the heart, thymus, lungs, liver, gallbladder, kidneys, pancreas, stomach, duodenum, jejunum, ileum, cecum, colon, lymph nodes (submandibular, mesenteric), salivary glands, skin (trunk and head), urinary bladder, uterus, cervix, vagina, ovaries, oviducts, adrenal glands, spleen, thyroid gland, esophagus, trachea, spinal cord, vertebrae, sternum, femur, tibia, stifle join, skeletal muscle, nerves, skull, nasal cavity, oral cavity, teeth, ears, eyes, pituitary gland, brain.

### Hematology

For hematology, blood was collected into tubes containing EDTA. Automated analysis was performed on an IDEXX Procyte DX hematology analyzer and the following parameters were determined: white blood cell count, red blood cell count, hemoglobin concentration, hematocrit, mean corpuscular volume, mean corpuscular hemoglobin, mean corpuscular hemoglobin concentration, red blood cell distribution width standard deviation and coefficient of variance, reticulocyte relative and absolute counts, platelet count, platelet distribution width, mean platelet volume, and relative and absolute counts of neutrophils, lymphocytes, monocytes, eosinophils, and basophils.

### Serum chemistry

For serum chemistry, blood was collected into tubes containing a serum separator, the tubes were centrifuged, and the serum was obtained for analysis. Serum chemistry was performed on a Beckman Coulter AU680 analyzer and the concentration of the following analytes was determined: alkaline phosphatase, alanine aminotransferase, aspartate aminotransferase, creatine kinase, gamma-glutamyl transpeptidase, albumin, total protein, globulin, total bilirubin, blood urea nitrogen, creatinine, cholesterol, triglycerides, glucose, calcium, phosphorus, chloride, potassium, and sodium. Finally, the Na/K ratio and albumin/globulin ratio were calculated.

### Isolation and immortalization of mouse embryonic fibroblasts

mEFs were isolated from E13.5 *Adam17Δcyto* embryos to generate primary mEFs as previously described (9, 71). Primary mEFs were immortalized by transduction with a retroviral plasmid carrying the simian virus 40 (SV40) large T antigen and a zeocin resistance cassette. The cell lines used in these studies were routinely genotyped by PCR to corroborate their identity.

### Western blot analysis

To generate western blots for ADAM9, ADAM17, and alpha tubulin with tissue extracts from adult mice, we harvested the brain, heart, lung, liver, and spleen samples from adult mice and processed the samples as previously described (66). Unless otherwise noted, all tissue and cell extracts were lysed on ice or at 4^o^C in phosphate-buffered saline (PBS) with 1% Triton-X 100, 5 μM Marimastat, 10 mM 1,10 Phenanthroline, and protease inhibitor cocktail (1:250) (48). For the ADAM17 and ADAM9 blots, it was crucial that the tissue or cell extract was subjected to Concanavalin A sepharose beads for glycoprotein enrichment (48). Comparable amounts of protein were separated under nonreducing conditions on 10% SDS-PAGE Tris-Glycine gels or on 10% Bis-Tris NuPage gels (Thermo Fisher Scientific, Cat#: NP0301), as indicated, and immobilized onto nitrocellulose membranes (Pall Corp, Cat. #: VMR 27376-991). After blocking in PBS, 0.05% Tween 20, and 5% nonfat dry milk for 1 h at room temperature, the nitrocellulose membrane was incubated overnight at 4 degrees with rabbit polyclonal antibodies raised against an Fc-fusion protein with the extracellular domain of ADAM17 that only recognizes nonreduced ADAM17. The membranes were then washed three times with PBS, 0.05% Tween 20, and incubated in HRP-conjugated goat anti-rabbit secondary (Promega). ADAM9 and alpha-tubulin were used as loading controls by running duplicate gels or by stripping the membranes for 15 min at 55^o^C in stripping buffer (2% SDS, 50 mM beta-mercaptoethanol in 62 mM Tris, pH 6.7). These membranes were blocked as described above, then incubated with anti-ADAM9 or anti-alpha-tubulin antibodies. Western blots for iRhom1, iRhom2, ubiquitin, and LC3 were performed on samples that were not enriched with Concanavalin A, as described previously (50).

### Biotinylation of cell surface proteins

Cell surface proteins of mEFs were labeled with 1 mg/ml of the membrane impermeable biotinylation reagent EZ-Link Sulfo-NHS-LC-Biotin (Thermo Fisher Scientific) following the manufacturer’s protocol. The cells were lysed in PBS, 1% Triton X-100 containing protease inhibitors including the metalloprotease inhibitors 1,10-phenanthroline (10 mM), and marimastat (5 μM). The biotinylated fraction of the lysate was pulled down with streptavidin-Sepharose 4B beads for 30 min at 4^o^C. The beads were washed four times with lysis buffer, and the bound material was eluted in SDS-containing sample loading buffer. The resulting material was then separated on a 10% SDS-PAGE gel and subjected to western blot analysis for ADAM17 or ADAM9 as described above. The ratio of cell surface biotinylated mature ADAM17 to total ADAM17 protein detected by western blot was determined from densitometric quantification of the bands by NIH Image J, normalized using ADAM9 as a loading control.

### Flow cytometry staining and ADAM17 MFI quantification

The skin was prepared for flow cytometry as previously described (72, 73). Murine ear skin was excised and finely minced and digested with dispase (Thermo Fisher Scientific), type II collagenase (Worthington) and DNAseI (Sigma Aldrich). Cells were then triturated with Pasteur pipettes and further incubated with EDTA before being passed through a 70 μm nylon filter to generate a single cell suspension used for flow cytometric staining.

Skin cells were stained with Fc-block (Biolegend) followed by anti-CD45 anti-EpCAM (all Biolegend) and anti-ADAM17. Donkey anti-rabbit IgG (Jackson Immunoresearch) was used to detect the anti-ADAM17. 4’,6-diamidino-2-phenylindole dihydrochloride (DAPI) was used to exclude dead cells and debris (Sigma Aldrich). Normal rabbit serum (Jackson Immunoresearch) was used as a negative control for ADAM17.

For ADAM17 mean fluorescence intensity (MFI) quantification, all ADAM17 MFIs were normalized to the wild-type control in the same experiment.

### TGFα ectodomain shedding assay

*Wild-type*, *Adam17Δcyto*, and *Adam17−/−* mEFs were transfected with alkaline phosphatase-tagged TGFα using Lipofectamine 2000, following the manufacturer’s instructions (Thermo Fisher, Cat. # 12566014). On the following day, the cells were incubated in Opti-MEM (Gibco) for 30 min and then fresh Opti-MEM was added for 45 min with or without the addition of 25 ng/ml phorbol 12-myristate 13-acetate (PMA) to stimulate ADAM17-dependent shedding, in the presence or absence of 5 μM marimastat. The supernatants were collected and the cells were lysed as described above. To measure the alkaline phosphatase activity in the cell supernatants and lysates, we added the alkaline phosphatase substrate para-nitrophenyl phosphate and measured the optical density at A_405nm_. The optical density was determined for three identical technical replicates, and the data shown represents three independent trials.

### Isolation of mouse primary bone-marrow-derived macrophages (BMDMs) and Csf1r flow cytometry

To generate primary BMDMs, we isolated bone marrows from adult mice (6 weeks or older with at least a 2:1 sex ratio distribution) as previously described (50). Bone marrow from femurs and tibiae was washed with PBS and passed through a 70 μm cell strainer (Denville Scientific) before plating on Petri dishes in DMEM supplemented with 20% fetal calf serum and 10 ng/ml of murine macrophage-colony stimulating factor (Peprotech, Cat. #: 315-02). After 7 days, macrophages were removed with Accutase (Sigma-Aldrich, Cat#: A6964), plated on tissue culture plates, and left untreated or treated with 25 ng/ml PMA for 45 min on the following day. BMDMs were lysed and subjected to western blot analysis for ADAM17 and alpha tubulin or the cell surface levels of the Csf1R were measured by flow cytometry with anti-CD115 antibodies (Biolegend, Clone: AFS98).

### qRT-PCR analysis

#### Tissues

Total RNA was isolated from the tissues (the brain, heart, lung, liver, spleen) of adult mice that were at least 6 weeks old using TRIzol reagent (Thermo Fisher Scientific). The resulting material was subjected to a standard ethanol/sodium acetate precipitation protocol for nucleic acids to remove salts and impurities and then used for reverse transcription, as outlined below.

#### Bone-marrow-derived macrophages

BMDMs were isolated as described above and differentiated for 7 days, at which point their RNA was isolated using the RNeasy mini kit from Qiagen.

Total RNA was reverse-transcribed using RNasin Plus (Promega), 2.5 mM dNTPs, Random Primer 9, and M-MuLV Reverse Transcriptase from NEB. ADAM17 oligonucleotides were purchased from Eurofins Genomics using previously described sequences (72), ADAM17 Forward 5’-GATGCTGAAGATGACACTGTG-3’ (A17 exon 14); ADAM17 Reverse 5’- GAGTTGTCAGTGTCAACGC-3’ (ADAM17 exon 14–15). GAPDH oligonucleotides were purchased from Qiagen. ADAM17 mRNA in *wild-type* and *Adam17Δcyto* tissues was quantified *via* RT-qPCR using SYBR Green Reagent and an ABI PRISM 7900HT cycler (both from Applied Biosystems, Thermo Fisher Scientific). GAPDH was used as a housekeeping control. Three independent experiments were performed with duplicate or triplicate samples for each experiment.

### TNFα ELISA

Primary BMDMs differentiated for 7 days (see above) were harvested using Accutase solution and re-plated at 1.25 × 10^5^ cells/well in 0.4 ml complete DMEM in a 24-well format. On the following day, BMDMs were treated with 2.0 μg/ml Lipopolysaccharide (LPS) for 3 h to stimulate production and shedding of TNFα or left untreated. After 3 h, the culture supernatants were collected to measure the amount of soluble released TNFα by ELISA with a kit specific for murine TNFα (Duoset ELISA DY410, R&D Systems).

### Cycloheximide chase assay and protein degradation inhibitors

*Wild-type* and *Adam17Δcyto* mEFs were plated in a 6-well format and grown to 90 to 100% confluency. Cells were then treated with 100 μg/ml cycloheximide in freshly prepared complete DMEM (10% FCS, 1% P/S) supplemented with 15 mM HEPES and lysed at the corresponding time points – 0 h, 0.5 h, 1 h, 2 h, and 4 h, as described above. The samples were enriched for glycoproteins using Concanavalin A sepharose beads and used for western blot analysis of ADAM17, with western blots of ADAM9 serving as loading control. The band intensities of wild-type ADAM17 and ADAM17Δcyto at each time point were determined *via* densitometry in ImageJ and used to calculate the percentage remaining at each time point compared with the levels at 0 h.

In western blot experiments with protein degradation inhibitors, the inhibitors were used at the following concentrations and time points: MG-132 and eeyarestatin at 10 μM for 6 h; Bafilomycin at 100 nM and chloroquine at 100 µM, both for 18 h (30, 31, 74).

### Statistical analysis

All graphs are presented as mean ± SEM and were analyzed with GraphPad Prism 8.4.3. To calculate the statistical significance, we proceeded as follows. An unpaired two-tailed Student’s *t*-test was used for the qRT-PCR results, AP-TGFα shedding assays, and ADAM17 MFI from flow cytometry experiments in Figure 5, Figure 6, Figure 7. A two-way ANOVA was used for the TNFα ELISA shown in Figure 9*E*. A Welch unpaired Student’s *t*-test and post hoc Bonferroni–Dunn correction for multiple hypothesis correction was used for the cycloheximide chase assay in Figure 10.

In all cases, a *p*-value or corrected *p*-value of <0.05 was considered statistically significant (indicated by ∗).

## Data availability

All the data described in this article are contained within the article.

## Supporting information

This article contains supporting information.

## Conflict of interest

Drs Maretzky and Blobel hold a patent on a method of identifying agents for combination with inhibitors of iRhoms. Dr Blobel and the Hospital for Special Surgery have identified iRhom2 inhibitors and have cofounded the start-up company SciRhom in Munich to commercialize these inhibitors.
