# Supplementary material for: Targeted truncation of the ADAM17 cytoplasmic domain in mice results in protein destabilization and a hypomorphic phenotype
Source: J Biol Chem. 2021 May 4;296:100733. doi: 10.1016/j.jbc.2021.100733 (PMC8191336; doi:10.1016/j.jbc.2021.100733)
Supplement: Supplemental Figures S1–S2 and Table S1 [file mmc1.pdf]

Supporting Data for the manuscript entitled "Targeted truncation of the ADAM17 cytoplasmic domain in mice results in strongly reduced protein levels and a hypomorphic phenotype", by Lora et al.

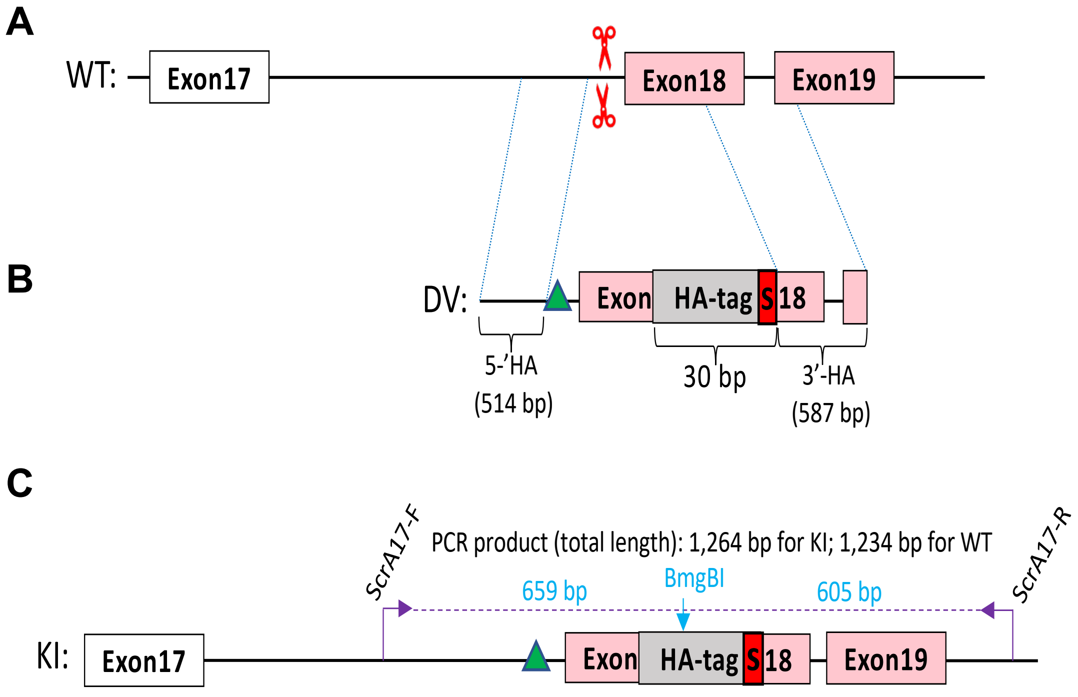

Supporting Data S1. Generation of the *Adam17Δcyto* mouse. (A) *Adam17 wild type (WT)* genomic DNA, showing exon 17, 18 and 19. The CRISPR/Cas9 guide chosen cleaves at the intron 17 region located 8 bp away from exon 18 (red scissors). (B) Donor vector (DV) carrying 5'-HA (homology arm), HA-tag, stop codon & 3'-HA in pFUSA vector backbone, serves as a repair template upon dsDNA break induced by Cas9 to introduce the KI mutant (HA-tag & stop codon, the latter labeled as 'S'). Green triangle indicates the 3 bp change made to prevent CRISPR from re-cutting. Blue dotted line spans the homology sequence used for DNA repair. (C) Genotyping for mESC carrying *Adam17Δcyto* delta KI was performed using PCR primer pair (ScrA17-F & ScrA17-R, see supplementary Table 1 for details) to amplify 1,264 bp for KI vs. 1,234 bp for WT. BmgBI restriction enzyme cleaves the 1,264 bp band into two fragments (659 bp and 605 bp) while the 1,234 bp remains intact.

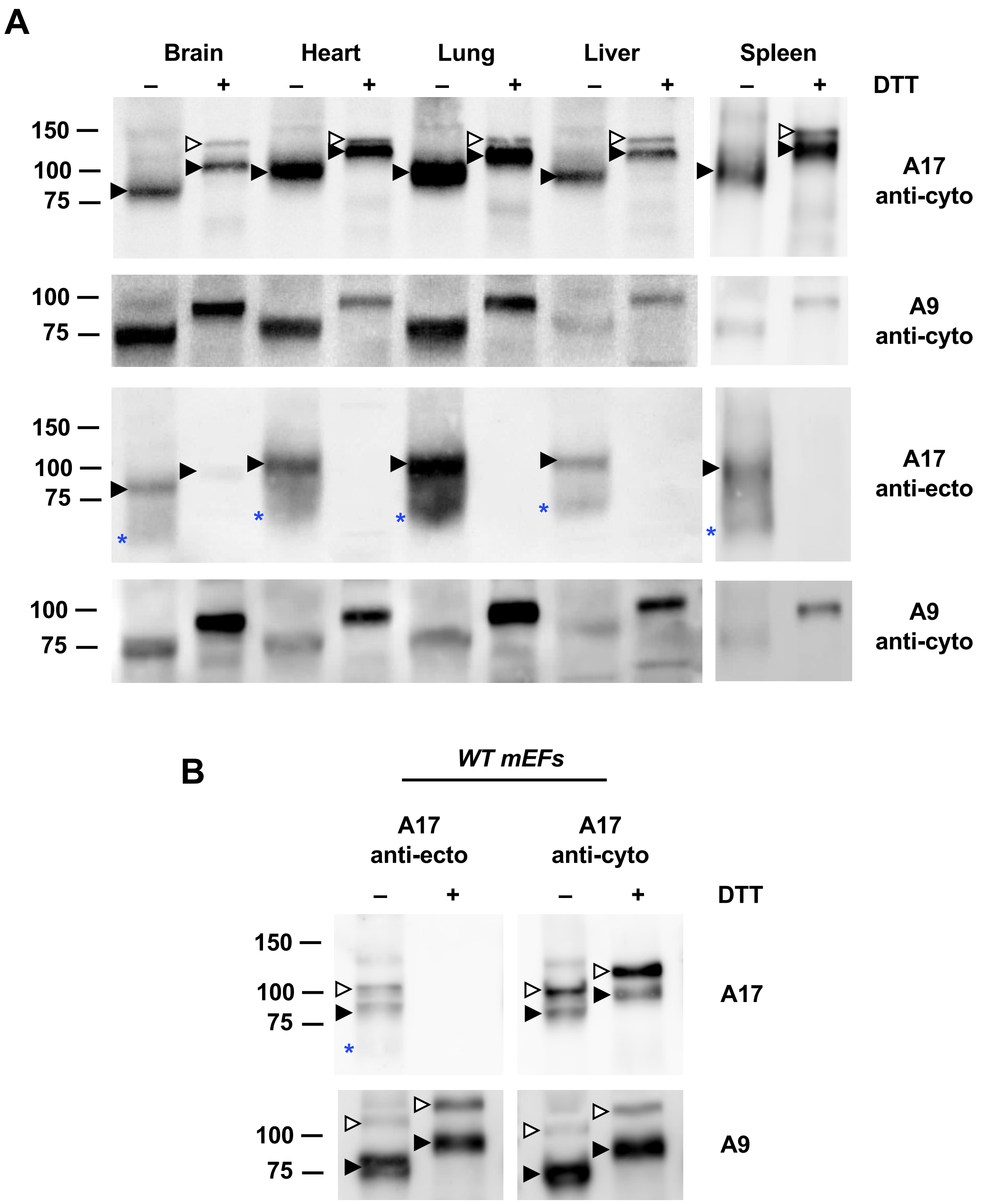

Supporting Data S2. ADAM17 Western blots adult *wild type* tissues under non-reducing and reducing conditions. A) Concanavalin-A enriched whole-tissue lysates of adult *wild type* mice were subjected to Western blot analysis and probed with rabbit polyclonal antibodies (pAbs) against the ADAM17 cytoplasmic domain or the anti-ADAM17-ecto pAbs on samples that were prepared under non-reducing or reducing conditions (boiled for 5 min in 50 mM DTT and then alkylated with 100 mM Iodoacetimide).

Under non-reducing conditions, the pro and mature form of ADAM17 of tissue samples are not resolved on a 10% SDS-PAGE gel (as shown by the Western blot using pAbs against the ADAM17 cytotail). The ADAM17-anti-ecto pAbs only recognize ADAM17 under non-reducing conditions and are able to bind to the form of ADAM17 that lacks its cytoplasmic domain due to a post-lysis autocatalytic degradation artifact. B) Western blots on *wild type* mEFs show that the anti-ADAM17-ecto antibodies detect the pro-form of non-reduced ADAM17 less efficiently than the anti-ADAM17-cyto antibodies, presumably because the pro-domain obscures epitopes in the mature protein until it is removed. The anti-ADAM17-ecto antibodies are unable to detect wild type ADAM17 under reducing conditions. Since mEFs and BMDM have significantly higher ratios of pro- to mature ADAM17 than tissues (see also (22,53)), this provides a likely explanation for why the anti-ADAM17-ectodomain antibodies detect the more abundant pro-form of ADAM17 in Western blots of mEFs and BMDM, but not in Western blots of different tissues, as shown in A). The blots were stripped and re-probed with anti-ADAM9 as a loading control. The results are representative of at least three independent experiments with separately isolated tissue samples.

Primer Name	Primer Sequence
5'-HA-F	TGACTTGGTCTCGCTATCCCTCTTGTTCTCCTGCA
5'-HA-R	CCCCTCGGTCTCGGCACGTCGTAGGGTAGTCCAGTTTCTTATCCTAGAAAGCATAGGAAG
3'-HA-F	GGACTAGGTCTCCGTGCCCGACTACGCC**TAG**AAGCAGTATGAATCCCTGTCTCTGTTTC
3'-HA-R	CTAACGGGTCTCTCGCCGGAAAGGGCTTGATGATGC
Mut-F	GAAAAAAAGAATGACATTTTTACTTCCTCATCTTTCTAGGATAAGAAACTGGACTACCC
ScrA17-F	GATGCTGTGAATAGTCACCTTTG
ScrA17-R	GGAAAGGGCTTGATGATGCG

Supplementary Table 1. Primers used for construction of the *Adam17Δcyto* donor vector (DV) and for genotyping. The BsaI restriction enzyme recognition site is underlined, the HA tag is indicated by a dotted line, the stop codon is bolded. The HA-tag and stop codon were sandwiched between the 5'-HA and 3'-HA and were PCR amplified from genomic DNA using two primer pairs (5'-HA-F, 5'-HA-R, 3'-HA-F, 3'-HA-R), followed by Golden Gate cloning for construction of the *Adam17Δcyto* DV. The Mut-F oligo was used to introduce the 3 bp mutagenesis, to prevent Cas9 re-cutting post recombination, using the QuikChange II site-directed mutagenesis kit (Agilent). The yellow highlight indicates the 3 bp mutated sequence. ScrA17-F & ScrA17-R were used for mESC genotyping.
